# Transient decline and early recovery of noninvasive myocardial work after kidney transplantation: a prospective study

**DOI:** 10.1080/0886022X.2025.2543930

**Published:** 2025-08-11

**Authors:** Yulin Huang, Lin Jin, Xinyan Qiu, Canying Yang, Luyi Ping, Zhengyao Jiang, Hua Zou, Zhihong Zhang, Jiwei Wang

**Affiliations:** a Department of Ultrasound, The Second Affiliated Hospital, Jiangxi Medical College, Nanchang University, Nanchang, Jiangxi, China; b Department of Transplantation, The Second Affiliated Hospital, Jiangxi Medical College, Nanchang University, Nanchang, Jiangxi, China; c School of Public Health, Jiangxi Medical College, Nanchang University, Jiangxi Provincial Key Laboratory of Disease Prevention and Public Health, Nanchang University, Nanchang, Jiangxi, China

**Keywords:** Chronic kidney disease, kidney transplantation, noninvasive myocardial work, left ventricular function

## Abstract

**Background:**

Patients with chronic kidney disease (CKD) are at a high risk of cardiovascular disease. This study aims to observe the short-term changes of left ventricular (LV) myocardial work in stage 5 CKD patients with successful kidney transplantation (KT).

**Methods:**

45 stage 5 CKD patients who are candidates for KT were enrolled. Changes in clinical variables, laboratory data, routine transthoracic echocardiography, and noninvasive myocardial work (NIMW) were analyzed at pre-KT, 10 days, and 3 months post-KT. NIMW parameters include global myocardial work index (GWI), global constructive work (GCW), global wasted work (GWW), and global work efficiency (GWE).

**Results:**

1) Renal function indicators, including blood urea nitrogen, serum creatinine, and estimated glomerular filtration rate improved significantly at 10 days post-KT. At 3 months post-KT, there appears to be a continuing recovery trend; 2) GWE, GWI, and GCW were significantly increased at 3 months post-KT, but GCW and GWI with an early decrease at 10 days post-KT; 3) At the 10 days post-KT, the changes in systolic blood pressure and hemoglobin were positively correlated with the changes in GWI. Meanwhile, the change in systolic blood pressure was also positively correlated with the change in GCW. The change in diastolic blood pressure was positively correlated with the change in GWW.

**Conclusion:**

LV systolic function doesn’t improve in parallel with renal function after a successful KT. Steadily controlling blood pressure and correcting anemia is associated with improving myocardial work after KT, especially in the early post-transplant period.

## Graphical Abstract

**Figure:**
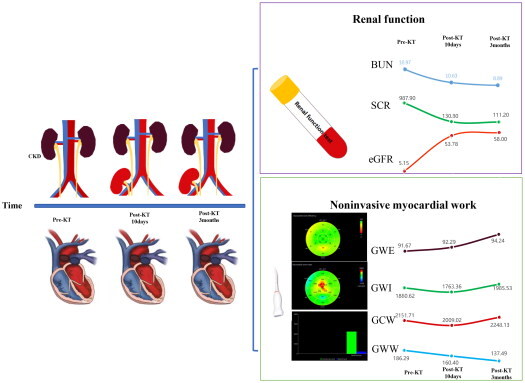

## Introduction

Chronic kidney disease (CKD) is a significant global health challenge, involving about 13.4% of the worldwide population [1]. CKD is highly related to cardiovascular diseases (CVD) for two main reasons: 1) Patients with CKD are at a high risk of CVD, including left ventricular (LV) remodeling and dysfunction, arrhythmia, coronary artery disease, and sudden cardiac death [2]; 2) CVD is the leading cause of death in CKD patients, approximately 40–50% of total mortality [3].

Kidney transplantation (KT), the most effective treatment for stage 5 CKD, could effectively improve renal function, prolong survival, reduce complications, and improve quality of life [4,5]. Some studies have shown that cardiac structure and function are improved with successful KT using various diagnostic techniques, including routine echocardiography, cardiopulmonary exercise testing, and speckle-tracking echocardiography [6–8]. However, the above studies have not provided data on short-term cardiac structure and function after KT. Is cardiac structure function rapidly improved following a successful KT? Is there a synchronous relationship between the restoration of cardiac function and the improvement of renal function? Currently, there is insufficient evidence to answer these questions.

A thorough understanding of short-term changes in cardiac function after KT may facilitate early cardiovascular risk stratification and management in this high-risk population. During the perioperative period, patients often receive fluid resuscitation and experience metabolic disturbances such as electrolyte imbalance and decreased hemoglobin levels [9,10]. These factors can significantly affect cardiac preload, afterload, and oxygen delivery, thereby directly influencing LV function. Early identification of cardiac functional changes may allow clinicians to optimize volume management strategies and adjust pharmacological interventions accordingly, potentially improving long-term cardiac outcomes.

Noninvasive myocardial work (NIMW) technology is a novel method for evaluating systolic cardiac function that accounts for deformation and afterload [11]. It has been considered more sensitive than left ventricular ejection function (LVEF) by routine echocardiography and global longitudinal strain (GLS) by speckle-tracking echocardiograph for detecting subclinical myocardial dysfunction [12–14]. This study aims: 1) to observe the short-term changes of LV structural and myocardial work in stage 5 CKD patients with successful KT; 2) to determine whether the cardiac function improves in parallel with renal function after successful KT.

## Materials and methods

### Study population

**Figure 1.**
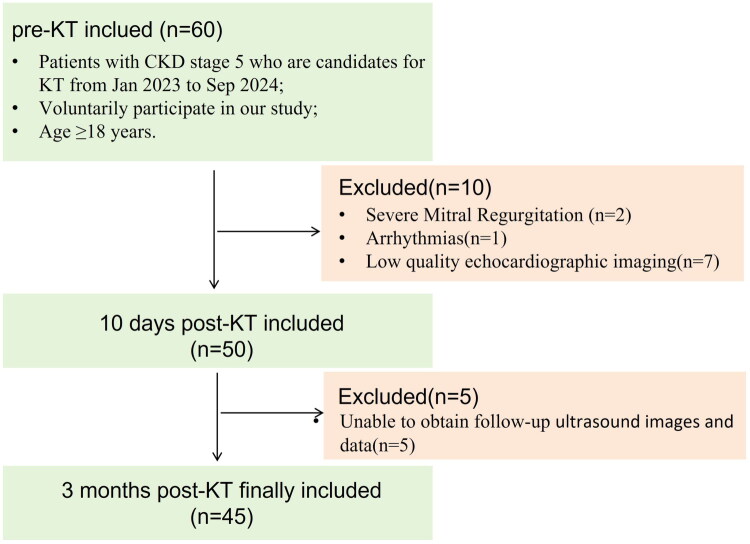
Flow chart of the study with inclusion and exclusion criteria. CKD, chronic kidney disease; KT, kidney transplantation.

It is a prospective cohort study approved by the Human Research and Ethics Committee of the Second Affiliated Hospital of Nanchang University. Inclusion criteria were as follows: (1) patients with stage 5 CKD who were candidates for KT between 2023 Jan and 2024 Sep; (2) voluntary participation in our study; (3) age ≥18 years. Exclusion criteria included: (1) other heart diseases, including congenital heart disease, valvular heart disease, or cardiomyopathy; (2) arrhythmias; (3) low-quality echocardiographic imaging; (4) inability to obtain follow-up data. According to the inclusion and exclusion criteria, 45 patients were eligible for the final analysis (Figure 1).

### Clinical and laboratory parameters

Demographic parameters such as age, gender, height, and weight were collected. Clinical variables, including heart rate (HR), systolic blood pressure (SBP), diastolic blood pressure (DBP), types of dialysis, and duration of dialysis, were gathered. Laboratory markers, containing B-type natriuretic peptide (BNP), blood urea nitrogen (BUN), serum creatinine (SCR), estimated glomerular filtration rate (eGFR), calcium (Ca), phosphorus (P), hemoglobin (HB), red blood cell (RBC), and C-reactive protein (CRP) were obtained from electronic medical records.

The formulas used for calculating body mass index (BMI), body surface area (BSA), and eGFR are as follows [15,16]: 1) BMI = Weight (kg)/Height(m)^2^; 2) BSA (m^2^) = (Height (cm) × Weight (kg)/3600) ^½^; 3) eGFR (ml/min/1.73 m^2^) = 175 × (serum creatinine in mg/dL) ^−1.154^ × (age in years) ^−0.203^ × (0.742 for women).

### Routine transthoracic echocardiography and GLS

**Figure 2.**
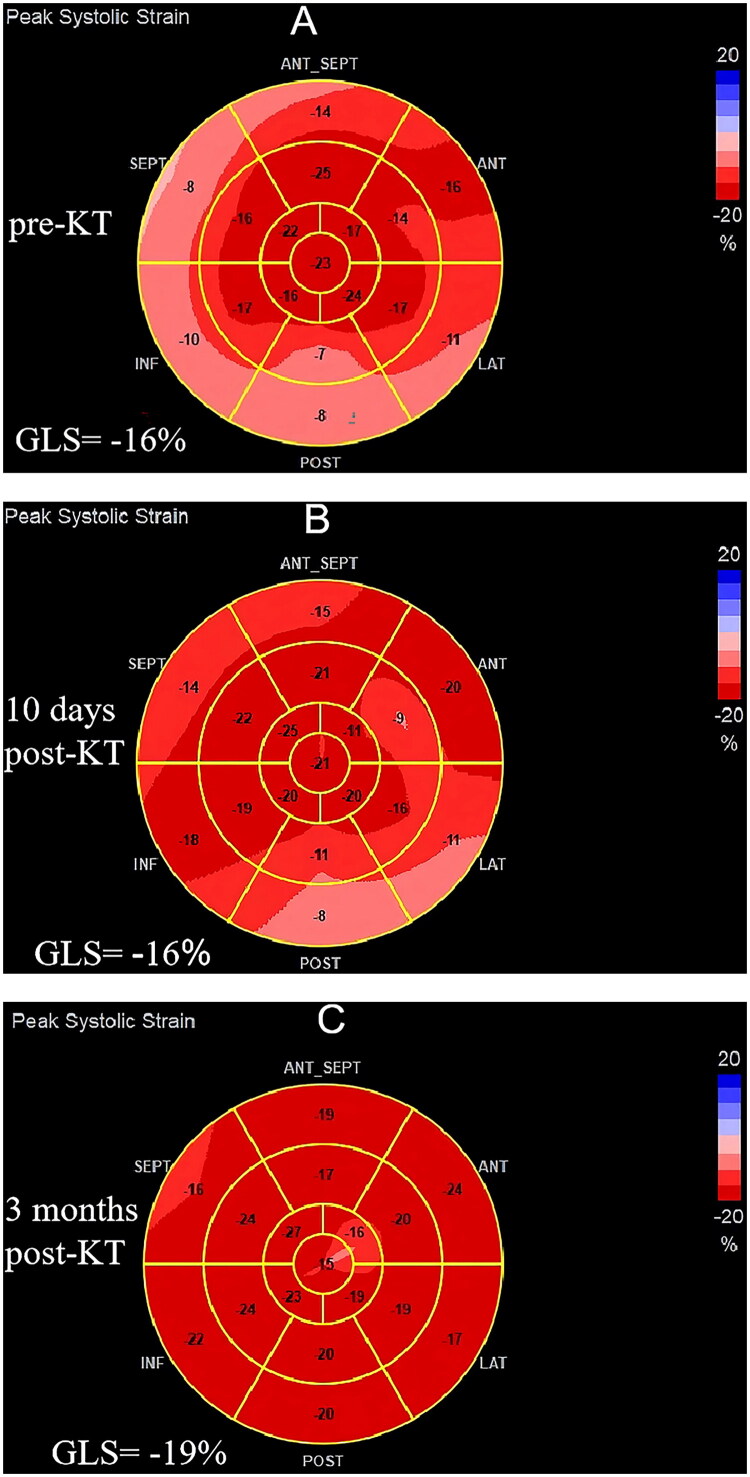
Case example of a CKD stage 5 patient showing GLS changes at pre-KT (a), 10 days post-KT (B), and 3 months post-KT (C). GLS, longitudinal strain; KT, kidney transplantation.

Transthoracic echocardiography (TTE) was conducted for each patient at pre-KT (on the day or the day before KT) and post-KT (about 10 days and 3 months after KT). All TTEs were performed by the same experienced physician using the Vivid E95 system (GE Healthcare) with an M5S transducer. Standard imaging windows and measurements were completed based on the current European Association of Echocardiography and American Society of Echocardiography guidelines [17]. Echocardiographic parameters included left ventricular posterior wall thickness (LVPWT), interventricular septal thickness (IVST), left ventricular end-diastolic diameter (LVEDD), left ventricular end-systolic diameter (LVESD), right ventricular end-diastolic diameter (RVEDD), left atrial anteroposterior diameter (LAAPD), and right atrial transverse diameter (RATD). LVEF was calculated using the biplane Simpson’s method. Left ventricular mass (LVM) was calculated using the Devereux formula: LVM(g) = 0.8{1.04[(LVEDD + LVPWT + IVST)^^3^–(LVEDD)^^3^]} + 0.6 [18], and left ventricular mass index (LVMI) = LVM/BSA [19]. In addition, images from the apical 4-chamber, 2-chamber, and 3-chamber views zoomed on the LV were acquired with a frame rate of ≥ 40 frames/sec to assess LV- GLS by speckle-tracking echocardiography (Figure 2).

### NIMW analysis

NIMW analysis was performed offline by the Echo PAC version 203 workstation (GE Healthcare). It was analyzed using a pressure-strain loop, calculated by integrating LV pressure curves and GLS. SBP from the brachial cuff was used to estimate peak LV systolic pressure. The LV pressure curve was generated automatically by software after setting the duration of isovolumic and ejection phases defined by valvular time events. The parameters of NIMW were defined as follows: global work index (GWI), global constructive work (GCW), global wasted work (GWW), and global work efficiency (GWE). GWE was calculated by GCW/(GCW + GWW) (Figure 3). All measurements and analyses were conducted by two experienced sonographers who were blinded to the patients’ time points of data acquisition.

Figure 3.Case example of a CKD stage 5 patient showing the change in myocardial work parameters at pre-KT, 10 days post-KT, and 3 months post-KT. GWI, global work index; GCW, global constructive work; GWW, global wasted work; GWE, global work efficiency; KT, kidney transplantation. (A-C), pre-KT; (D-F), 10D post-KT; (G-I), 3 M post-KT.

**Figure:**
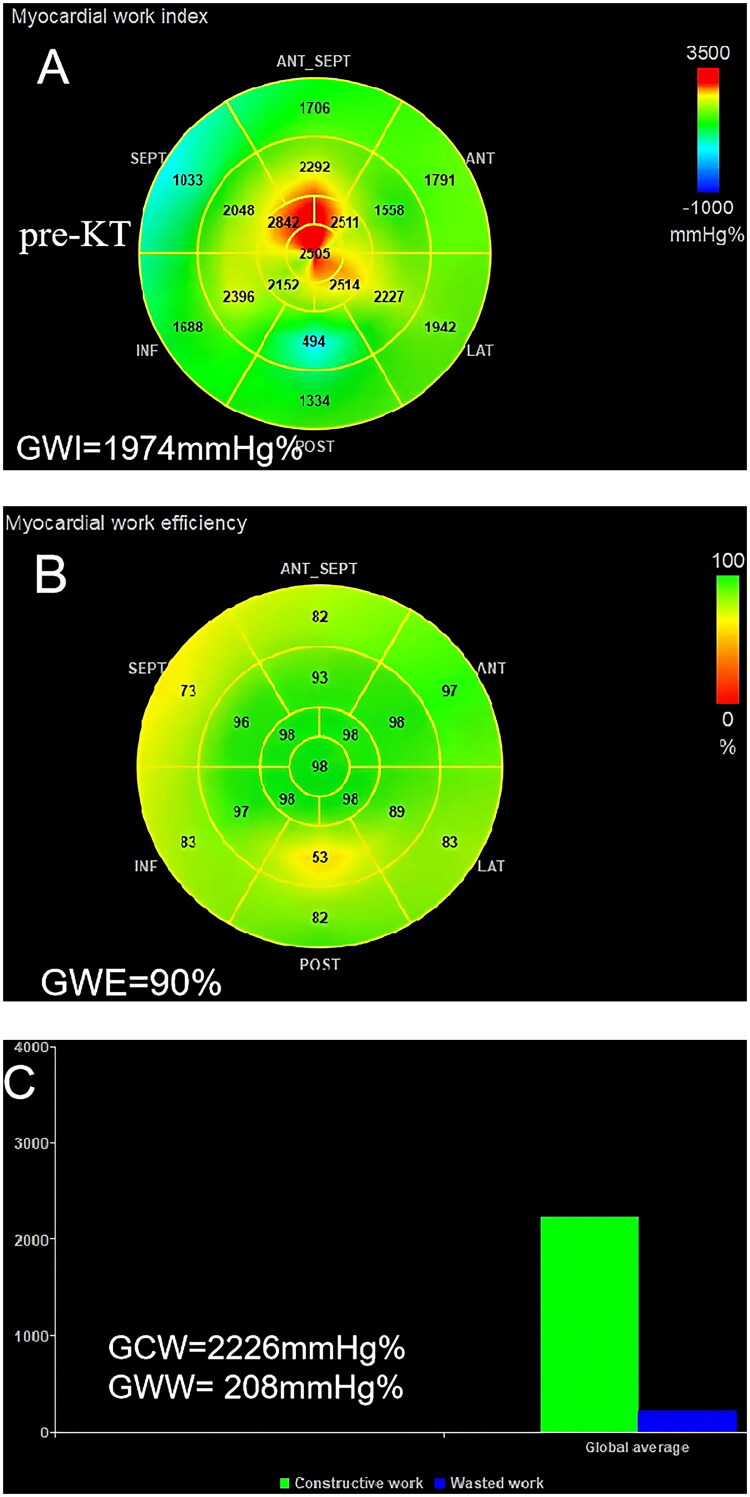

**Figure:**
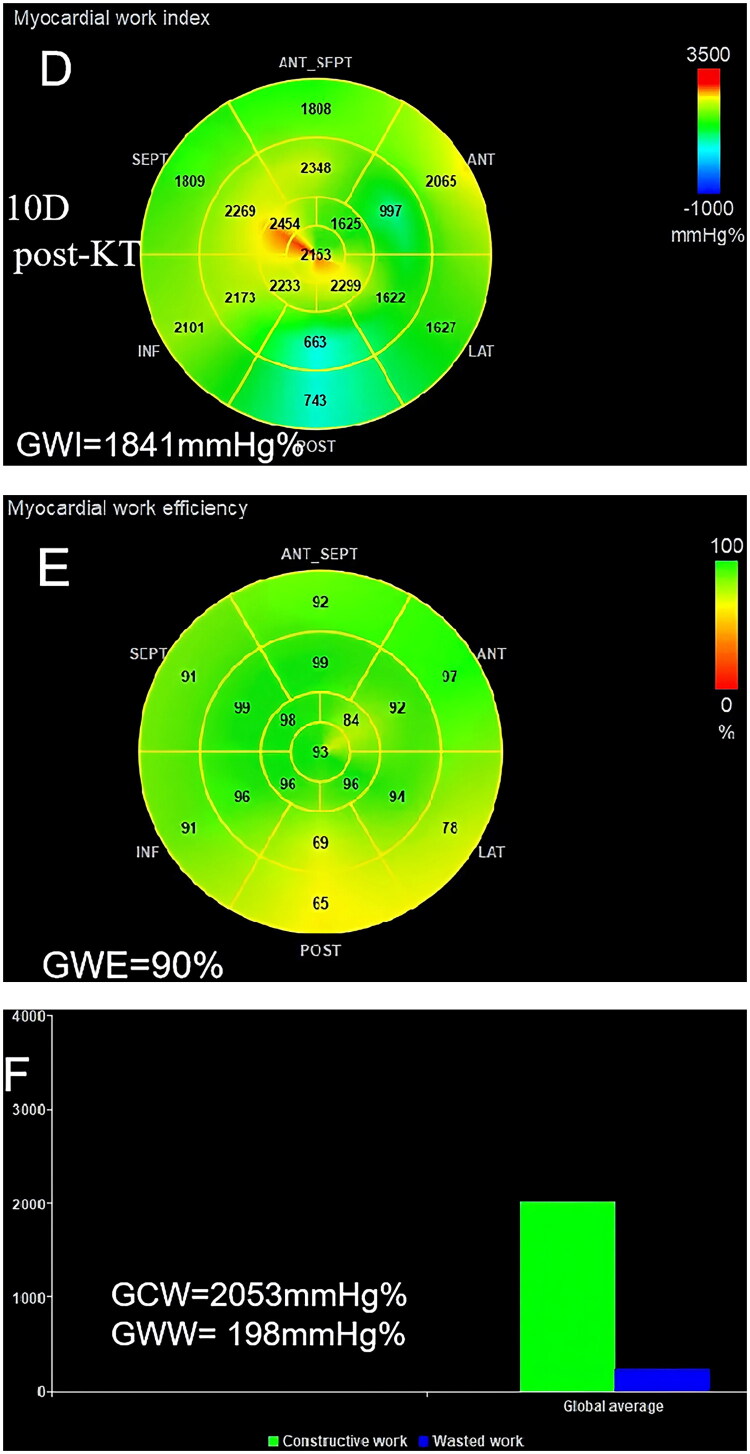

**Figure:**
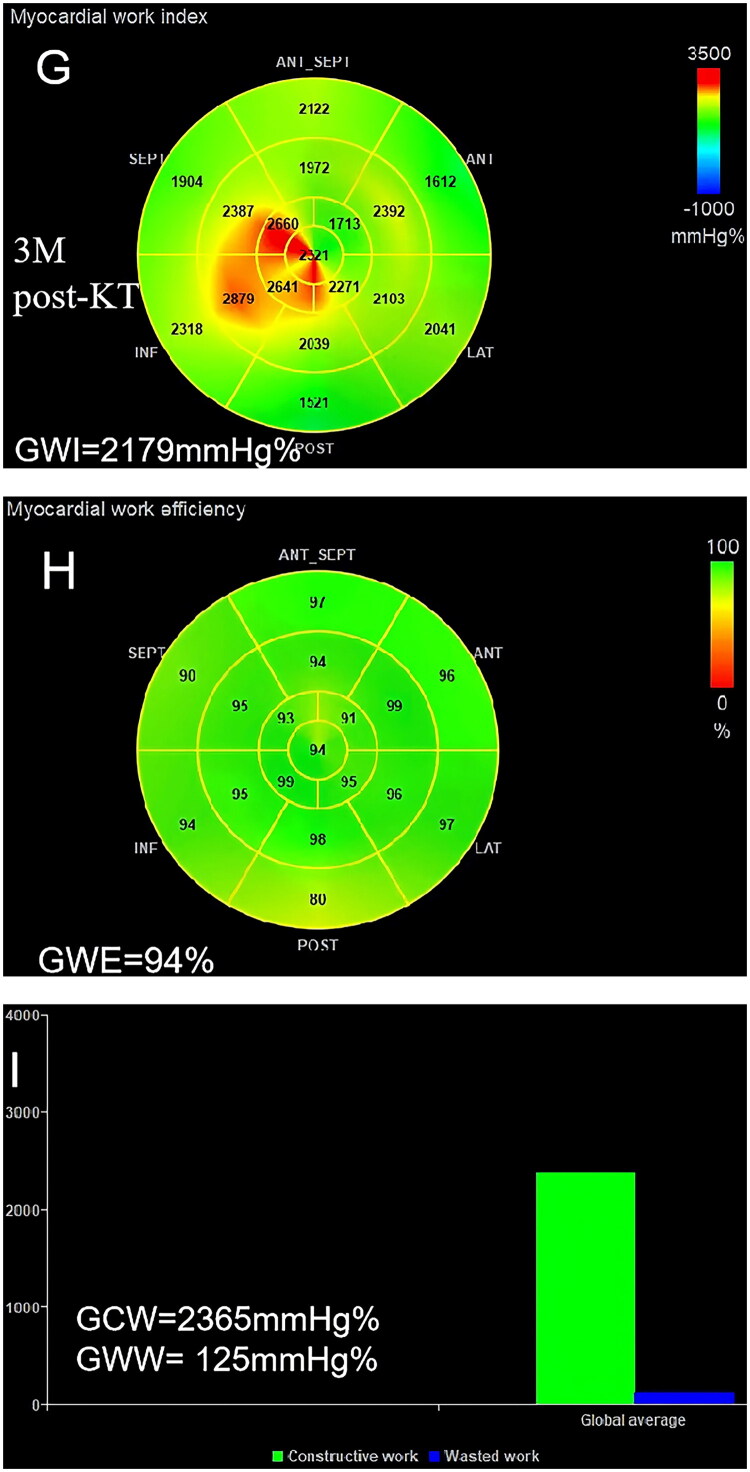

### Statistical analysis

Statistical analysis was conducted using SPSS software version 27.0. In this study, approximately 206 statistical tests were performed. Continuous variables were summarized as mean ± standard deviations if normally distributed, and as median (interquartile range) if not. Categorical variables were described as frequencies (percentages). For normally distributed continuous variables, one-way ANOVA was conducted, followed by Tukey’s Honestly Significant Difference (HSD) *post hoc* test for equal variances or Tamhane’s T2 for unequal variances (with adjusted *p* -values). For non-normally distributed data, the Kruskal-Wallis test was applied, and pairwise comparisons were performed using Bonferroni correction. Categorical variables were analyzed using the Fisher exact test or Chi-square test. The Pearson or Spearman correlation coefficients were applied to evaluate the linear relationship between the NIMW parameters and renal function indicators. Univariate and multivariate linear regression analyses were performed to identify the correlation between the changes in clinical variables and the NIMW parameters. A *p*-value less than 0.05 was considered statistically significant. Additionally, a post-hoc power analysis was performed using PASS 2021 software, based on the observed effect sizes of key NIMW parameters (GWE, GWI, GCW, and GWW) derived from paired comparisons between pre-transplant and 3-month post-transplant data.

## Results

### General clinical characteristics

**Figure 4.**
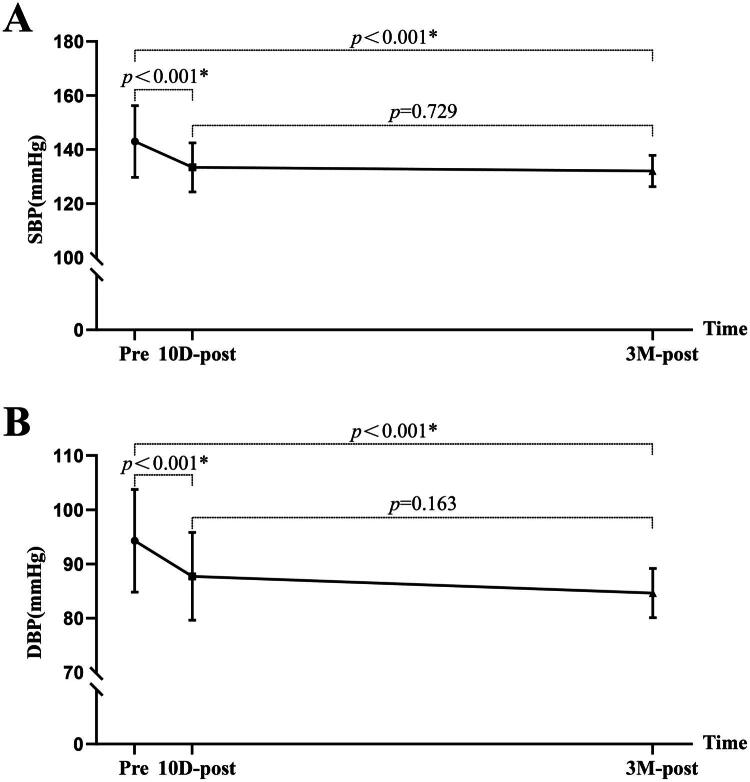
The comparison of SBP and DBP changes among pre-KT,10 days post-KT, and 3 months post-KT. SBP, systolic blood pressure; DBP, diastolic blood pressure; pre, pre-KT; 10D-post, 10 days post-KT; 3 M-post, 3 months post-KT.

A total of 45 CKD stage 5 patients with successful KT were eligible for final analysis after inclusion and exclusion screenings (mean age 44.09 ± 10.09 years; 64.4% male). The general clinical characteristics among pre-KT, 10 days post-KT, and 3 months post-KT are shown in Table 1. All patients received dialysis (71.1% hemodialysis, 28.9% peritoneal dialysis) before KT with a median time on dialysis of 2 (IQR: 1.00–4.00) years. Hypertension, anemia, and hyperphosphatemia were observed in 93.3, 73.3, and 97.7% of patients before KT, respectively. Compared to pre-KT, SBP (142.33 ± 10.60 v. 133.49 ± 9.67 mmHg, *p* < 0.001) and DBP (94.16 ± 9.68 v. 87.71 ± 8.01 mmHg, *p* < 0.001) decreased significantly at 10 days post-KT, and showed a continually downward trend at 3 months post-KT (Figure 4).

**Table 1.** The comparison of general clinical characteristics among pre-KT, 10 days post-KT, and 3 months post-KT.

Parameters	Pre	10D-Post	3M-post	*p* value		
	(*n* = 45)	(*n* = 45)	(*n* = 45)	Pre vs 10D-Post	Pre vs 3 M-Post	10D vs 3 M-Post
Male (%)	29 (64.4)	29 (64.4)	29 (64.4)	/	/	/
Age (years)	44.09 ± 10.09	44.09 ± 10.09	44.09 ± 10.09	/	/	/
Dialysis type (hemo/peritoneal)	45 (32/13)	0	0	/	/	/
Dialysis duration (years)	2.00 (1.00 ∼ 4.00)	/	/	/	/	/
Hypertension (%)	42 (93.3)	42 (93.3)	42 (93.3)	/	/	/
Anemia (%)	33 (73.3)	42 (93.3)	26 (57.8)	0.011*	0.120	<0.001*
Hyperphosphatemia (%)	44 (97.7)	1 (2.2)	0 (0)	<0.001*	<0.001*	0.315
BSA (m^2^)	1.65 ± 0.14	1.64 ± 0.15	1.63 ± 0.14	0.834	0.775	0.994
BMI (kg/m^2^)	21.74 ± 2.67	21.45 ± 2.67	21.20 ± 2.58	0.865	0.603	0.895
HR (bpm)	79.58 ± 11.33	81.89 ± 9.07	76.07 ± 6.45	0.204	0.398	0.009*
SBP (mmHg)	142.33 ± 10.60	133.49 ± 9.67	132.04 ± 6.23	<0.001*	<0.001*	0.729
DBP (mmHg)	94.16 ± 9.68	87.71 ± 8.01	84.64 ± 5.58	<0.001*	<0.001*	0.163

Data presented as mean (standard deviation) or median (interquartile range) for continuous variables and as frequencies (percentages) for categorical variables. BSA, body surface area; BMI, body mass index; SBP, systolic blood pressure; DBP, diastolic blood pressure; HR, heart rate; KT, kidney transplantation; *: represents *p* value < 0.05.

### Laboratory parameters

The laboratory parameters among pre-KT, 10 days post-KT, and 3 months post-KT are shown in Table 2.

**Table 2.** The comparison of the laboratory parameters among pre-KT, 10 days post-KT, and 3 months post-KT.

Parameters	Normal reference values	Pre	10D-Post	3M-post	*p* value		
		(*n* = 45)	(*n* = 45)	(*n* = 45)	Pre vs 10D -Post	Pre vs 3M -Post	10D vs 3M -Post
BUN (mmol/L)	2.1–7.1	18.97(16.40– 26.22)	10.63(8.89– 15.85)	8.89(7.40– 11.79)	<0.001*	<0.001*	0.065
SCR (umol/L)	57–110	987.90(753.85–1181.35)	130.80(84.55–202.95)	111.20(90.70– 161.95)	<0.001*	<0.001*	0.433
eGFR (mL/min/1.73 m²)	≥90	5.15 ± 1.37	53.78 ± 24.60	58.00 ± 19.53	<0.001*	<0.001*	0.749
Ca (mmol/L)	2.1–2.6	2.28 ± 0.25	2.24 ± 0.20	2.40 ± 0.15	0.726	0.027*	<0.001*
P (mmol/L)	0.81–1.45	1.75 ± 0.56	0.75 ± 0.33	0.80 ± 0.24	<0.001*	<0.001*	0.836
HB (g/L)	110–150(F) 130–175(M)	111.00(101.00–117.50)	90.00(82.50–103.50)	119.00(109.00– 126.00)	<0.001*	0.022*	<0.001*
RBC (10^12^/L)	3.8–5.1(F) 4.3–5.8(M)	3.69(3.49– 3.99)	3.10(2.90– 3.53)	4.01(3.71– 4.26)	<0.001*	0.015*	<0.001*
BNP (pg/mL)	0–100	75.00(42.80– 143.50)	99.00(78.34–151.50)	47.00(36.00– 70.00)	0.034*	0.006*	<0.001*
CRP (mg/L)	5–10	3.84 ± 1.86	4.16 ± 2.04	2.75 ± 1.22	0.664	0.010*	<0.001*

Data presented as mean (standard deviation) or median (interquartile range) for continuous variables. BUN, blood urea nitrogen; SCR, serum creatinine; eGFR, estimated glomerular filtration rate; Ca, calcium; P, phosphorus; HB, hemoglobin; RBC, red blood cell; BNP, B-type natriuretic peptide; KT, kidney transplantation; CRP, C-reactive protein; *: represents *p* value < 0.05.

Renal function indicators, including BUN (18.97[16.40–26.22] v. 10.63[8.89–15.85] mmol/L, *p* < 0.001), SCR (987.90[753.85–1181.35] v. 130.80[84.55–202.95] mmol/L, *p* < 0.001), and eGFR (5.15 ± 1.37 v. 53.78 ± 24.60 mL/min/1.73 m^2^, *p* < 0.001) improved significantly at 10 days post-KT. There appears to be a continuing recovery trend at 3 months post-KT, but still a little short of the normal reference range (Figure 5A–C).

Figure 5.The comparison of the laboratory parameters among pre-KT, 10 days post-KT, and 3 months post-KT. BUN, blood urea nitrogen; SCR, serum creatinine; eGFR, estimated glomerular filtration rate; Ca, calcium; P, phosphorus; HB, hemoglobin; RBC, red blood cell; BNP, B-type natriuretic peptide; pre, pre-KT; 10D-post, 10 days post-KT; 3 M-post, 3 months post-KT.

**Figure:**
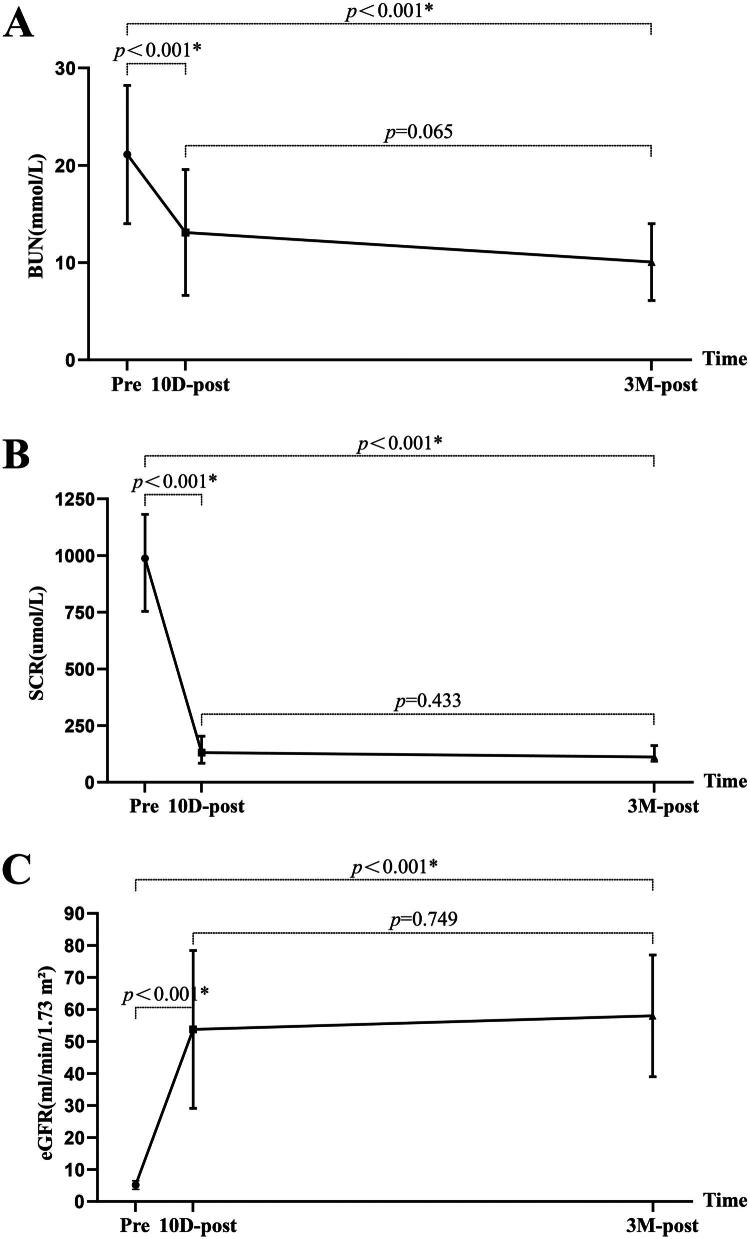

**Figure:**
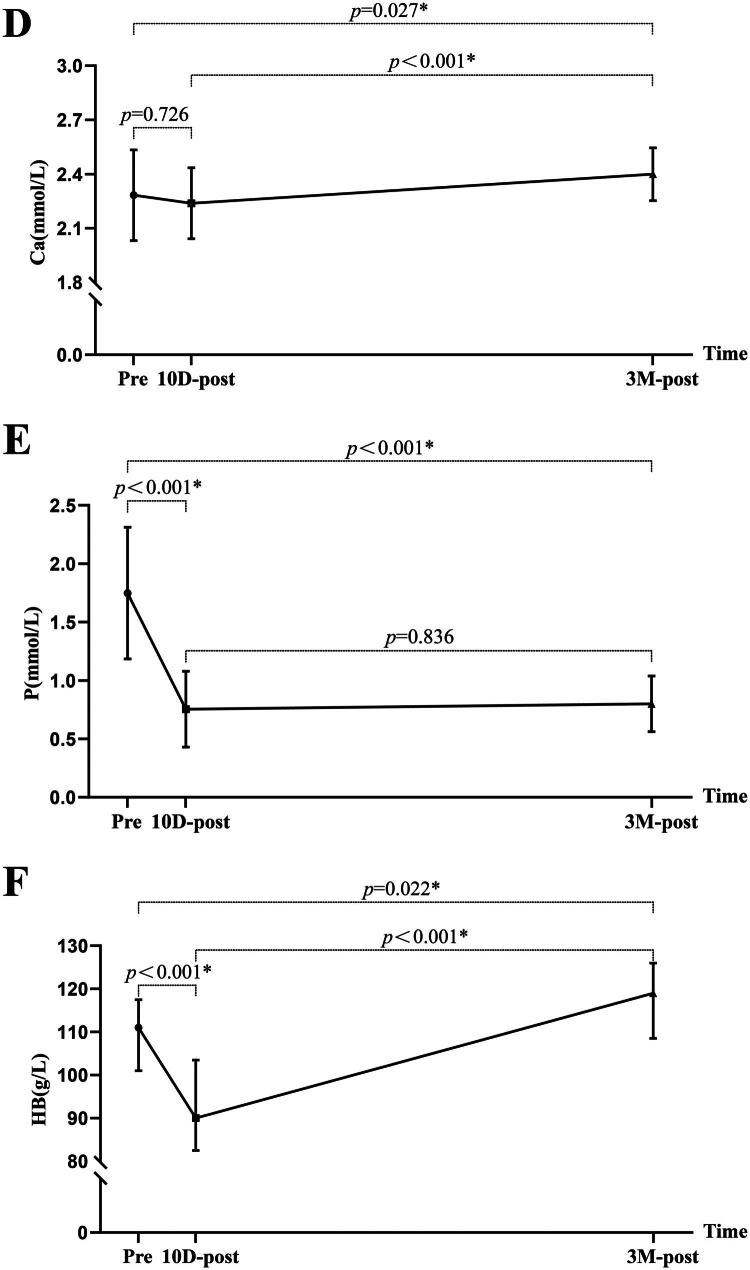

**Figure:**
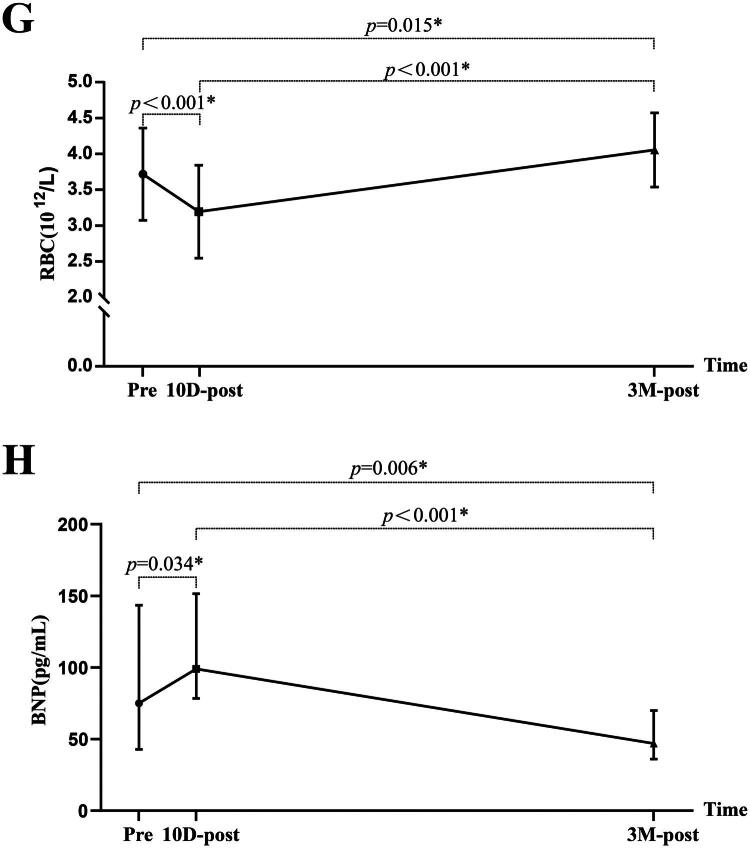

Serum Ca did not change at 10 days post-KT but increased significantly at 3 months post-KT. Serum P significantly decreased at 10 days post-KT, with no significant change between 10 days and 3 months post-KT (Figure 5D–E). Both HB and RBC decreased significantly at 10 days post-KT but increased by 3 months post-KT (Figure 5F–G). BNP shows a slight increase trend at 10 days post-KT (75.00[42.80–143.50] v. 99.00[78.34–151.50 mmol/L], *p* = 0.034), but a significant decrease (75.00[42.80–143.50] v. 47.00[36.00–70.00] mmol/L, *p* = 0.006) was demonstrated at 3 months post-KT (Figure 5H). Although CRP levels decreased at 3 months post- KT compared to pre-KT levels, all measurements remained within the normal range both before and after KT (3.84 ± 1.86 v. 2.75 ± 1.22 mg/L, *p* = 0.010).

### Routine transthoracic echocardiography parameters and GLS

Routine echocardiographic parameters and GLS among pre-KT, 10 days post-KT, and 3 months post-KT are listed in Table 3. No apparent changes were present in LAAPD, RATD, RVEDD, IVST, and LVPWT at either 10 days or 3 months post-KT. LVEDD, LVESD, LVMI, LVEF, and GLS did not show obvious alterations at 10 days post-KT. However, LVEDD (47.32 ± 4.31 v. 45.30 ± 3.92 mm, *p* = 0.048), LVESD (30.81 ± 3.03 v. 28.48 ± 5.02 mm, *p* = 0.011), and LVMI (111.67 ± 24.29 v. 100.80 ± 20.25 g/m^2^, *p* = 0.047) decreased significantly, whilst LVEF (58.40 ± 2.90% v.60.67 ± 3.41, *p* = 0.003) and absolute value of GLS (−16.96 ± 1.77 v.-18.31 ± 1.76%, *p* < 0.001) increased significantly 3 months post-KT.

**Table 3.** The comparison of routine transthoracic echocardiography parameters and GLS among pre-KT, 10 days post-KT and 3 months post-KT.

Parameters	Pre	10D-Post	3M-post	*p* value		
	(*n* = 45)	(*n* = 45)	(*n* = 45)	Pre vs 10D -Post	Pre vs 3 M -Post	10D vs 3 M -Post
LAAPD (mm)	33.96 ± 4.67	33.24 ± 3.68	32.58 ± 3.03	0.656	0.210	0.690
RATD (mm)	33.71 ± 3.56	33.20 ± 2.73	32.53 ± 2.24	0.680	0.134	0.520
RVEDD (mm)	21.27 ± 1.76	20.96 ± 1.54	20.67 ± 1.28	0.604	0.158	0.647
IVST (mm)	11.11 ± 1.16	10.92 ± 1.11	10.88 ± 1.16	0.707	0.591	0.981
LVPWT (mm)	10.31 ± 1.12	10.21 ± 1.09	10.01 ± 1.09	0.909	0.407	0.662
LVEDD (mm)	47.32 ± 4.31	47.11 ± 4.21	45.30 ± 3.92	0.970	0.048*	0.092
LVESD (mm)	30.81 ± 3.03	30.34 ± 2.87	28.48 ± 5.02	0.824	0.011*	0.054
LVEF (%)	58.40 ± 2.90	58.47 ± 3.27	60.67 ± 3.41	0.995	0.003*	0.004*
LVMI (g/m^2^)	111.67 ± 24.29	110.07 ± 23.87	100.80 ± 20.25	0.941	0.047*	0.135
GLS (%)	−16.96 ± 1.77	−16.89 ± 1.72	−18.31 ± 1.76	0.982	<0.001*	<0.001*

Data presented as mean (standard deviation) or median (interquartile range) for continuous variables. LAAPD, left atrial anteroposterior diameter; RATD, right atrial transverse diameter; RVEDD, right ventricular end-diastolic diameter; IVST, interventricular septal thickness; LVPWT, left ventricular posterior wall thickness; LVEDD, left ventricular end-diastolic diameter; LVESD, left ventricular end-systolic diameter; LVEF, left ventricular ejection fraction; LVMI, left ventricular mass index; GLS, longitudinal strain; KT, kidney transplantation; *: represents *p* value < 0.05.

### NIMW parameters

NIMW parameters among pre-KT, 10 days post-KT, and 3 months post-KT are presented in Table 4. Compared to pre-KT, GWI (1880.62 ± 178.48 v. 1763.36 ± 141.36 mmHg%, *p =* 0.001), GCW (2151.71 ± 189.23 v. 2009.02 ± 137.74 mmHg%, *p* < 0.001), and GWW (186.29 ± 51.11 v. 160.40 ± 40.80 mmHg%, *p =* 0.028) were significantly decreased at 10 days post-KT. At 3 months post-KT, GWE (91.67 ± 1.71 v. 94.24 ± 1.57%, *p* < 0.001), GWI (1880.62 ± 178.48 v. 1985.53 ± 137.99 mmHg%, *p =* 0.004), and GCW (2151.71 ± 189.23 v. 2248.13 ± 126.48 mmHg%, *p* = 0.017) were significantly increased, but GWW (186.29 ± 51.11 v. 137.49 ± 34.05 mmHg%, *p* < 0.001) showed a continuous decrease (Figure 6).

Figure 6.The comparison of myocardial work parameters among pre-KT,10 days post-KT, and 3 months post-KT. GWI, global work index; GCW, global constructive work; GWW, global wasted work; GWE, global work efficiency; pre, pre-KT; 10D-post, 10 days post-KT; 3 M-post, 3 months post-KT.

**Figure:**
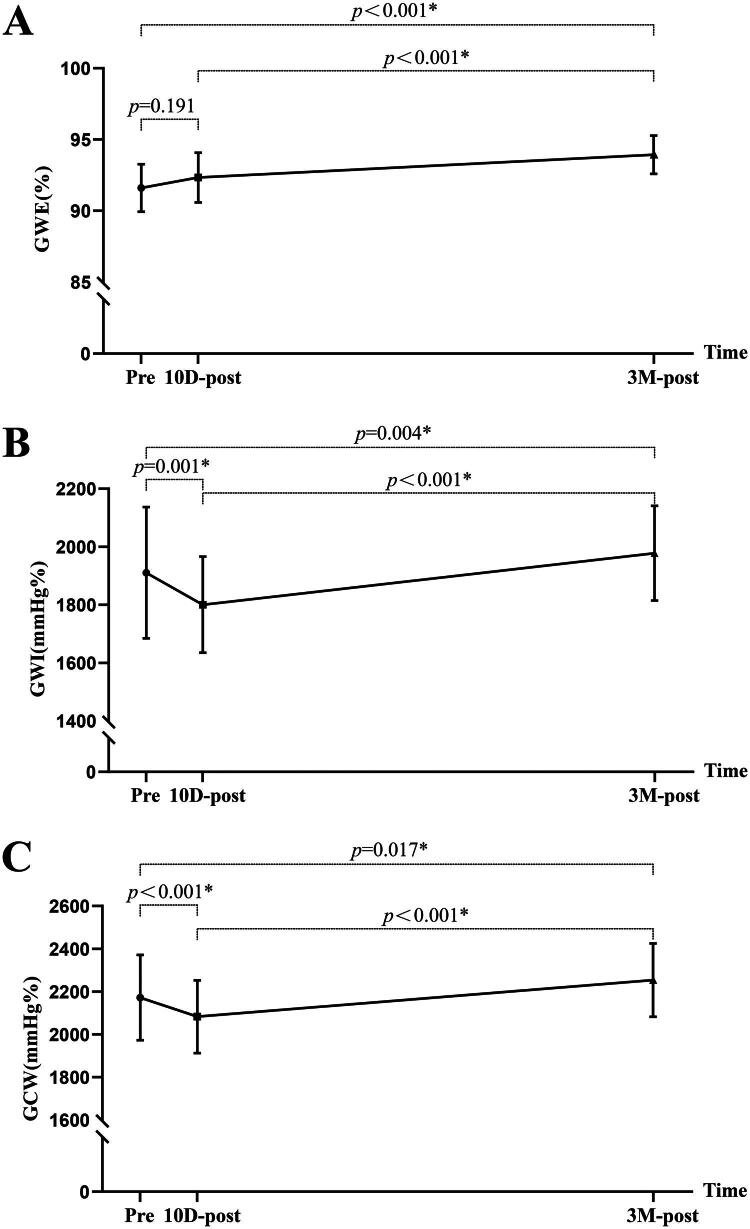

**Figure:**
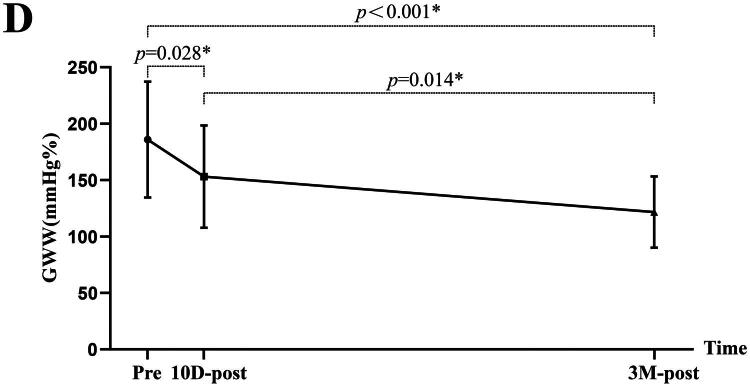

**Table 4.** The comparison of NIMW parameters among pre-KT,10 days post-KT, and 3 months post-KT.

Parameters	Pre	10D-Post	3M-post	*p* value		
	(*n* = 45)	(*n* = 45)	(*n* = 45)	Pre vs 10D -Post	Pre vs 3 M -Post	10D vs 3 M - Post
GWE (%)	91.67 ± 1.71	92.29 ± 1.78	94.24 ± 1.57	0.191	<0.001*	<0.001*
GWI (mmHg%)	1880.62 ± 178.48	1763.36 ± 141.36	1985.53 ± 137.99	0.001*	0.004*	<0.001*
GCW (mmHg%)	2151.71 ± 189.23	2009.02 ± 137.74	2248.13 ± 126.48	<0.001*	0.017*	<0.001*
GWW (mmHg%)	186.29 ± 51.11	160.40 ± 40.80	137.49 ± 34.05	0.028*	<0.001*	0.014*

Data presented as mean (standard deviation) or median (interquartile range) for continuous variables. GWI, global work index; GCW, global constructive work; GWW, global wasted work; GWE, global work efficiency; KT, kidney transplantation; *: represents *p* value < 0.05.

### Correlations between renal function indicators and NIMW parameters

Correlations between renal function indicators and NIMW parameters among pre-KT,10 days post-KT, and 3 months post-KT are displayed in Table 5. At pre-KT and 10 days post-KT, there was little correlation observed between renal function indicators and NIMW parameters, except for BUN, which was negatively correlated with GWI (*r* = −0.429, *p* = 0.003). and GCW (*r* = −0.327, *p* = 0.013) at pre-KT. At 3 months post-KT, eGFR showed a negative correlation with GWW (*r* = −0.396, *p* = 0.007) and a positive correlation with GWE (*r* = 0.429, *p* = 0.003). BUN showed negative correlations with GWI (*r* = −0.443, *p* = 0.002) and GWE (*r* = −0.432, *p* = 0.003), but a positive correlation with GWW (*r* = 0.375, *p* = 0.011). SCR showed negative correlations with GWI (*r* = −0.373, *p* = 0.012), GCW (*r* = −0.320, *p* = 0.032), and GWE (*r* = −0.415, *p* = 0.005), but a positive correlation with GWW (*r* = 0.348, *p* = 0.019).

**Table 5.** The correlation between renal function indicators and myocardial work parameters among pre-KT,10 days post-KT and 3 months post-KT.

	Variables	GWI		GCW		GWW		GWE	
		*r*	*p*	*r*	*p*	*r*	*p*	*r*	*p*
Pre -KT	eGFR	−0.013	0.913	−0.003	0.982	−0.188	0.215	0.160	0.293
	BUN	−0.429	0.003*	−0.327	0.013*	0.087	0.571	−0.291	0.052
	SCR	0.032	0.834	−0.022	0.887	0.016	0.919	0.001	0.994
10D-Post KT	eGFR	0.157	0.302	0.176	0.247	−0.149	0. 328	0.226	0.136
	BUN	−0.148	0.332	−0.036	0.816	0.156	0.306	−0.190	0.212
	SCR	−0.161	0.289	−0.060	0.696	0.088	0.564	−0.134	0.381
3M-Post KT	eGFR	0.292	0.052	0.170	0.265	−0.396	0.007*	0.429	0.003*
	BUN	−0.443	0.002*	−0.280	0.062	0.375	0.011*	−0.432	0.003*
	SCR	−0.373	0.012*	−0.320	0.032*	0.348	0.019*	−0.415	0.005*

BUN, blood urea nitrogen; SCR, serum creatinine; eGFR, estimated glomerular filtration rate; GWI, global work index; GCW, global constructive work; GWW, global wasted work; GWE, global work efficiency; KT, kidney transplantation.

### Univariate and multiple linear regression analysis of changes in NIMW parameters

Univariate and multiple linear regression analysis of changes in NIMW parameters are summarized in Tables 6 and 7. Multiple regression analysis showed that: 1) At the 10 days post-KT, the changes in SBP (*β* = 0.440, *p* = 0.019) and HB (*β* = 0.415, *p* = 0.008) were positively correlated with the changes in GWI. Meanwhile, the change in SBP (*β* = 0.551, *p* = 0.005) was also positively correlated with the change in GCW. Only the change of DBP (*β* = 0.431, *p* = 0.044) was correlated positively and independently with the alternation of GWW. 2) At the 3 months post-KT, the change of BUN (*β* = −0.286, *p* = 0.039) was positively correlated with the alternation of GWI. Furthermore, the changes in DBP (*β* = −0.432, *p* = 0.023) and SCR (*β* = *−*0.331, *p* = 0.025) were correlated negatively with the changes in GCW. Additionally, the change of HB (*β* = −0.531, *p* = 0.008) was correlated negatively with the alternation of GWW, while the changes in eGFR (*β* = 0.323, *p* = 0.017) and HB (*β* = 0.476, *p* = 0.008) were correlated positively with the changes of GWE.

**Table 6.** Univariate and multiple liner regression analysis of changes of NIMW parameters at 10 days post-KT.

	△GWI		△GCW		△GWW		△GWE	
Univariate	*r*	*p*	*r*	*p*	*r*	*p*	*r*	*p*
△SBP	0.411	0.005*	0.455	0.002*	0.159	0.296	−0.037	0.808
△DBP	−0.092	0.546	0.308	0.029*	0.386	0.005*	−0.283	0.060
△BUN	−0.259	0.086	−0.252	0.094	−0.134	0.380	−0.049	0.751
△SCR	0.059	0.702	−0.075	0.623	−0.197	0.194	−0.155	0.309
△eGFR	0.099	0.517	0.149	0.328	0.068	0.658	0.083	0.588
△ P	0.098	0.521	−0.031	0.842	0.033	0.827	−0.032	0.834
△HB	0.397	0.007*	0.274	0.069	−0.156	0.306	0.287	0.056
△RBC	0.058	0.703	0.041	0.791	−0.085	0.577	0.151	0.323
△CRP	0.012	0.937	−0.064	0.674	−0.020	0.897	0.050	0.744
Multiple	β	*p*	β	*p*	β	*p*	β	*p*
△SBP	0.440	0.019*	0.551	0.005*	−0.054	0.795	0.195	0.368
△DBP	0.014	0.938	0.035	0.846	0.431	0.044*	−0.417	0.058
△BUN	−0.289	0.087	−0.227	0.184	−0.146	0.453	−0.013	0.947
△SCR	−0.094	0.502	−0.243	0.094	−0.213	0.196	0.070	0.677
△eGFR	−0.007	0.962	0.019	0.901	−0.028	0.875	0.144	0.434
△ P	0.171	0.252	−0.002	0.991	0.080	0.642	−0.015	0.933
△HB	0.415	0.008*	0.244	0.113	−0.049	0.779	0.081	0.651
△RBC	−0.123	0.384	−0.074	0.606	−0.099	0.545	0.150	0.376
△CRP	−0.032	0.822	−0.141	0.331	−0.021	0.900	0.071	0.674

△, symbol of changes. SBP, systolic blood pressure; DBP, diastolic blood pressure; BUN, blood urea nitrogen; SCR, serum creatinine; eGFR, estimated glomerular filtration rate; P, phosphorus; HB, hemoglobin; RBC, red blood cell; GWI, global work index; GCW, global constructive work; GWW, global wasted work; GWE, global work efficiency; KT, kidney transplantation; CRP, C-reactive protein.

**Table 7.** Univariate and multiple liner regression analysis of changes of NIMW parameters at 3 months post-KT.

Univariate	△GWI		△GCW		△GWW		△GWE	
	*r*	*p*	*r*	*p*	*r*	*p*	*r*	*p*
△SBP	−0.338	0.023*	−0.297	0.047*	0.068	0.658	−0.226	0.136
△DBP	−0.326	0.029*	−0.444	0.002*	0.023	0.881	−0.166	0.227
△BUN	−0.325	0.029*	−0.155	0.310	−0.029	0.851	−0.078	0.609
△SCR	−0.304	0.042*	−0.396	0.007*	−0.242	0.110	−0.155	0.309
△eGFR	0.162	0.287	0.025	0.813	−0.380	0.010*	0.467	0.001*
△ P	0.243	0.108	0.098	0.520	−0.026	0.864	0.185	0.223
△HB	0.459	0.002*	0.232	0.126	−0.471	0.001*	0.508	<0.001*
△RBC	0.221	0.144	0.101	0.509	−0.062	0.688	0.042	0.783
**△**CRP	−0.382	0.010*	−0.230	0.129	0.216	0.155	−0.347	0.020*
Multiple	*β*	*p*	*β*	*p*	*β*	*p*	*β*	*p*
△SBP	−0.052	0.766	0.122	0.518	0.006	0.974	−0.068	0.694
△DBP	−0.158	0.355	−0.432	0.023*	−0.036	0.846	−0.033	0.843
△BUN	−0.286	0.039*	−0.135	0.354	−0.050	0.734	−0.087	0.514
△SCR	−0.224	0.095	−0.331	0.025*	−0.178	0.226	0.143	0.278
△eGFR	0.056	0.674	0.027	0.849	−0.270	0.070	0.323	0.017*
△ P	0.222	0.103	0.050	0.729	−0.076	0.608	0.225	0.096
△HB	0.191	0.275	0.062	0.741	−0.531	0.008*	0.476	0.008*
△RBC	−0.003	0.984	−0.125	0.435	0.179	0.279	−0.248	0.097
**△**CRP	−0.275	0.072	−0.215	0.186	−0.086	0.603	−0.058	0.692

△, symbol of changes. SBP, systolic blood pressure; DBP, diastolic blood pressure; BUN, blood urea nitrogen; SCR, serum creatinine; eGFR, estimated glomerular filtration rate; P, phosphorus; HB, hemoglobin; RBC, red blood cell; GWI, global work index; GCW, global constructive work; GWW, global wasted work; GWE, global work efficiency; CRP, C-reactive protein.

### Reproducibility of the NIMW parameters

To assess the reproducibility of the ultrasound parameter measurements in our study, echocardiographic images from 8 randomly selected patients were reanalyzed. Intraclass correlation coefficients (ICCs) along with 95% confidence intervals (CIs) for each parameter are presented in Table 8. The results demonstrated excellent reproducibility of the NIMW parameters, with ICC values exceeding 0.75 for both intra-observer and inter-observer assessments.

**Table 8.** Inter- and intra-observer variability of the NIMW parameters.

Parameters	Inter-observer		intra-observer	
	ICC	95%CI	ICC	95%CI
GWE	0.908	0.610-0.981	0.917	0.643-0.983
GWI	0.957	0.803-0.991	0.977	0.892-0.995
GCW	0.949	0.768-0.990	0.954	0.789-0.991
GWW	0.913	0.629-0.982	0.915	0.636-0.982

ICC, intraclass correlation coefficient; GWI, global work index; GCW, global constructive work; GWW, global wasted work; GWE, global work efficiency.

## Discussion

Accurate assessment of cardiac function is crucial for guiding patient management and determining prognosis. LVEF is a widely accepted method for assessing LV function in most guidelines but is unsuitable for detecting subclinical changes in systolic function [20]. GLS by speckle tracking echocardiography is more reproducible than LVEF regardless of echocardiographic training [21], and more sensitive than LVEF in detecting early subclinical myocardial dysfunction [22]. The NIMW technology was a novel method for evaluating systolic cardiac function. It incorporates deformation and load into its analysis, making it superior to LVEF and GLS in assessing subclinical myocardial dysfunction.

In this study, we used the NIMW technique to compare LV systolic function changes for CKD stage 5 patients before and after KT treatment. Almost all patients had generally preserved LVEF before and after KT, and no apparent differences in LVEF and GLS until 3 months after KT. These results are consistent with previous studies reporting early stabilization of cardiac function: Hamidi et al. [23] used two-dimensional speckle-tracking echocardiography and found no significant improvement in GLS one month after KT. Similarly, Khani et al. [24] assessed right ventricular function using conventional transthoracic echocardiography from one week to three months postoperatively and reported no notable changes in LVEF. In contrast, our study observed an improvement in LVEF at three months post-KT, suggesting that LV systolic function may begin to recover in the early to intermediate postoperative period. However, most NIMW parameters, including GWI, GCW, and GWW, displayed significant changes 10 days after KT. Our study is the first to demonstrate a significant reduction in myocardial work at 10 days post-KT, a time point that has been scarcely explored in the context of cardiac functional adaptation. This finding provides further support for previous studies that the NIMW technique is more sensitive than LVEF and GLS for the detection of subclinical myocardial dysfunction.

CVD is highly prevalent in patients with CKD, and subsequent CKD is a significant risk factor for CVD. As the optimal treatment for stage 5 CKD, KT can reverse some of the CVD. Prior studies have demonstrated KT can reduce cardiac mortality and decrease the risk of development of heart failure [25,26]. Some echocardiographic studies have reported reduced LVM and positive changes in LV systolic function (as determined by (LVEF, GLS, and NIMW) following KT [8,10, 27,28]. Most studies, however, have not described the short-term effects following KT. Our study focused on this issue and made the following findings: 1) LVEDD, LVESD, LVMI, LVEF, and GLS did not improve significantly until 3 months post-KT; 2) BNP showed a significant decrease at 3 months post-KT with a brief early rise at 10 days post-KT; 3) GWI, GCW, and GWW were significantly decreased at 10 days post-KT. At 3 months post-KT, GWE, GWI, and GCW were significantly increased, but GWW showed a continuous decrease. Our findings are partially consistent with those of Han et al. [29], who also evaluated changes in MW parameters before and after KT in patients with uremia and reported significant improvements one year after surgery. However, our study demonstrated that such improvements could already be observed at 3 months post-transplantation, suggesting that cardiac remodeling and functional recovery may begin earlier than previously recognized. Based on the above results, we can conclude that the reversal of LV structure and function is time-dependent, a finding corroborated by Jhinger et al. [30] independent observations lend further support to our conclusion. Notably, there is a transient decrease in LV systolic function in the early post-transplantation period (detected at 10 days post-KT). However, LV structure and systolic function are improved during the short-term follow-up (detected at 3 months post-KT).

Restoration of renal function after KT disrupts the negative cardiorenal interplay reverses the changes in cardiac structure and function. Is there a synchronous relationship between the restoration of cardiac function and the improvement of renal function? In the present study, we made the following findings: 1) Different from BNP and NIMW parameters, renal function indicators, including BUN, SCR, and eGFR, improved significantly at 10 days post-KT. 2) At pre-KT and 10 days post-KT, little correlation was observed between renal function indicators and NIMW parameters, except for BUN negatively related to GWI at pre-KT. At 3 months post-KT, eGFR showed a negative correlation with and a positive correlation with GWE. BUN showed negative correlations with GWI and GWE, but a positive correlation with GWW. SCR showed negative correlations with GWI, GCW, and GWE, but a positive correlation with GWW. From this data, we can conclude that cardiac function does not improve synchronously with renal function in the early post-transplantation period.

CKD leads to CVD due to several factors, including hypertension, fluid overload, uremic toxins, anemia, mineral metabolism disorder, and arteriovenous fistulas [31,32]. Vice versa, the recovery of cardiac structure and function after KT may be explained by several mediating factors, including the clearance of uremic toxins, control of BP, resolution of anemia, correction of hyperphosphatemia, and reversal of fluid overload [32–35]. In the present study, we also observed some of the above factors, and made the following findings: 1) 93.3% of patients with hypertension before KT. SBP and DBP decreased significantly at 10 days post-KT and showed a continually downward trend at 3 months post-KT. 2) Renal function indicators, including BUN, SCR, and eGFR improved significantly at 10 days post-KT. There appears to be a continuing recovery trend at 3 months post-KT, but still a little short of the normal reference range. 3) 73.3% of patients with anemia before KT. HB decreased significantly at 10 days post-KT but increased by 3 months post-KT; 4) 97.7% of patients with hyperphosphatemia before KT. Serum P decreased significantly at 10 days post-KT, with no significant change between 10 days and 3 months post-KT; 5) no significant difference in BMI among pre-KT, 10 days post-KT, and 3 months post-KT. According to the above results, we speculate that the clearance of uremic toxins, control of BP, resolution of anemia, and correction of hyperphosphatemia may associate with the change of cardiac structure and function in the short-term after KT. However, there is absent evidence to support the idea that the reversal of fluid overload is associated with the changes in cardiac structure and function after KT.

To investigate the factors leading to the changes in LV systolic function (as determined by NIMW parameters) after KT, we analyzed the relationship between changes in NIMW parameters and clinical variables by univariate and multiple linear regression analysis. Our findings are as follows: 1) At the 10 days post-KT, the changes in SBP and HB were positively correlated with the changes in GWI. Meanwhile, the change in SBP was also positively correlated with the change in GCW. Only the change of DBP was correlated positively and independently with the alternation of GWW. 2) At the 3 months post-KT, the change of BUN was negatively correlated with the alternation of GWI. Furthermore, the changes in DBP and SCR were correlated negatively with the changes in GCW. Additionally, the change in HB was correlated negatively with the alternation of GWW, while the changes in eGFR and HB were correlated positively with the changes in GWE. The above results indicate that in the early stage after KT, the reduction in NIMW parameters appears to be primarily associated with decreased BP and HB. However, at the short-term follow-up, the restoration in NIMW parameters results from decreased BP and SCR and increased HB and eGFR. These findings suggest that, beyond improving renal function, maintaining optimal BP and HB levels may aid in recovering cardiac structure and function in the short term after KT.

The observed reduction in NIMW during the early postoperative period after KT can be explained by several interrelated mechanisms:1) Reduction in BP. Myocardial work is a comprehensive index reflecting the interaction between myocardial deformation and left ventricular pressure, primarily influenced by BP [36]. The significant reduction in BP after KT indicates decreased LV afterload and peripheral resistance. Myocardial contractile function may remain impaired or still be adapting following prolonged uremic exposure in the 10 days following KT. 2) Iron deficiency. Prior studies have shown that early anemia after KT is primarily attributed to iron deficiency, resulting from surgical blood loss and frequent post-transplant blood tests, rather than incomplete renal function recovery [37]. Firstly, iron deficiency can reduce collagen synthesis in myocardial cells, which alters the pressure-volume relationship and reduces the elasticity of the myocardium [38]. Secondly, iron deficiency can lead to myocardial ischemia and hypoxia symptoms, impaired energy metabolism and mitochondrial biosynthesis, and disorder of intracellular ion homeostasis, resulting in cell edema, loss of normal contract-relaxation ability [39]. 3) Postoperative stress response and ischemia-reperfusion injury. Postoperative pain can activate the hypothalamic-pituitary-adrenal (HPA) axis, leading to elevated serum cortisol levels and sustained sympathetic nervous system activation, resulting in increased catecholamine release [40]. Oxidative metabolites of catecholamines can interfere with calcium handling in cardiomyocytes, causing excitation–contraction coupling imbalance and cardiotoxicity, thereby impairing myocardial function [41]. During the KT period, ischemia-reperfusion injury further exacerbates oxidative stress, which damaging mitochondrial function and cardiomyocyte structure, which leads to temporary cardiac dysfunction [42]. 4) Perioperative fluid management and myocardial edema. Postoperative volume expansion supports graft perfusion [9], but rapid fluid shifts during tapering may cause transient hemodynamic instability and myocardial stunning, impairing early myocardial performance [43]. Cardiac magnetic resonance studies have shown that interstitial myocardial edema can persist up to 8 weeks after KT in patients with stage 5 CKD [44]. This edema may impair myocardial relaxation and deformation, thus negatively impacting myocardial work. 5) Immunosuppressive therapy. Immunosuppressants prevent rejection but may cause cardiotoxicity. In particular, calcineurin inhibitors (such as cyclosporine and tacrolimus), are closely associated with cardiotoxicity and vasoconstriction. These drugs may impair vascular endothelial function and elevate BP, thereby increasing LV afterload and compromising myocardial energy metabolism. Moreover, they can activate pro-fibrotic pathways, including transforming growth factor-β, leading to interstitial collagen deposition in the myocardium, increased myocardial stiffness, and reduced contractile efficiency [45]. For clarity, the aforementioned proposed mechanisms and corresponding literature support are summarized in Table 9.

**Table 9.** Summary of potential mechanisms for early postoperative decline in NIMW after KT.

Mechanism	Pathophysiological explanation	Authors and References
Reduction in blood pressure (afterload unloading)	A reduction in systemic blood pressure decreases the left ventricular pressure–strain relationship, resulting in lower myocardial work values.	Russell et al. [36]
Iron deficiency	Iron deficiency impairs mitochondrial function, collagen synthesis, and intracellular ion homeostasis, ultimately culminating in impaired contractile function.	Chvapil et al. [38]
		Elçioğlu et al. [10]
Postoperative stress response and Ischemia-reperfusion injury	The stimulation of the hypothalamic-pituitary-adrenal axis induces a pronounced surge in catecholamines, resulting in myocardial dysfunction and calcium mishandling, and potentially leading to adrenergic-mediated cardiotoxicity.	Adameova et al. [40]
		Ivascu et al. [41]
		Kumar et al. [42]
Fluid overload and myocardial edema	Excessive preload and acute volume fluctuations can induce myocardial stunning or ischemia, potentially leading to hemodynamic compromise and adverse cardiovascular outcomes. Interstitial edema affects myocardial relaxation and deformation, leading to a reduction in early post-transplant myocardial work.	Guaricci et al. [43] Hayer et al. [44]
Immunosuppressive therapy	Immunosuppressive therapy is associated with fibrosis, endothelial dysfunction, and cardiotoxicity, thereby increasing afterload and reducing myocardial efficiency.	Opałk et al. [45]

This study has some limitations. Firstly, as a single-center study with a relatively small sample size, there is a potential for selection bias, and the small sample size also limits the statistical power, especially for detecting small to moderate effect sizes. Future studies involving larger, multi-center cohorts are warranted to validate these findings, particularly during the early post-transplant period. Secondly, this was a short-term follow-up study. In our study period, the renal function indicators were still slightly short of the normal reference range. Thirdly, some potentially important time-varying factors, such as minor infections, medication adjustments, and fluid shifts, were not fully accounted for in our analysis, which may introduce bias in evaluating the association between KT and myocardial outcomes. Last but not least, our study explores the short-term changes of LV structural and myocardial work before and after KT, the underlying mechanisms behind these changes require further exploration.

In conclusion, this study implied that LV systolic function may lag behind renal function recovery after a successful KT. The renal function rapidly recovers, while LV myocardial work slightly decreases in the early stage after KT. However, LV structure and systolic function are improved during the short-term follow-up. Steadily controlling BP and correcting anemia is associated with cardiac structure and function after KT, especially in the early post-transplant period. Since the NIMW technique is more sensitive than LVEF and GLS at detecting subclinical myocardial dysfunction, NIMW parameters can be utilized to closely monitor myocardial function, allowing for adjustments in treatment strategies to facilitate recovery after KT.

## Data Availability

The data underlying this article are available in the article and in its online supplementary material.
